# Investigation of HCAR2 antagonists as a potential strategy to modulate bovine leukocytes

**DOI:** 10.1186/s40104-024-00999-5

**Published:** 2024-03-06

**Authors:** Laman K. Mamedova, Kirby C. Krogstad, Paiton O. McDonald, Laxman Pokhrel, Duy H. Hua, Evan C. Titgemeyer, Barry J. Bradford

**Affiliations:** 1 Department of Animal Science, Michigan State University, East Lansing, Michigan 48824 USA; 2 Department of Animal Sciences and Industry, Kansas State University, Manhattan, KS 66506 USA; 3 Comparative Medicine and Integrative Biology, East Lansing, MI 48824 USA; 4 Department of Chemistry, Kansas State University, Manhattan, KS 66506 USA

**Keywords:** Flow cytometry, GPR109A, HM74A, Ketosis

## Abstract

**Background:**

Dairy cows experiencing ketosis after calving suffer greater disease incidence and are at greater risk of leaving the herd. In vitro administration of beta-hydroxybutyric acid (BHBA; the primary blood ketone) has inhibitory effects on the function of bovine leukocytes. BHBA is a ligand of HCAR2 and the activation of these receptors promotes an anti-inflammatory response which may be related with immunosuppression observed in transition dairy cattle. The objective of this study was to identify and test antagonists for HCAR2 in bovine immune cells cultured with BHBA.

**Results:**

We observed expression of HCAR2 at the protein level within lymphocytes, monocytes, and granulocytes. The proportion of cells expressing HCAR2 tended to be greater in mid-lactation compared to early lactation cows; the increase was a result of increased proportion of T and B cells expressing HCAR2. Stimulation of HCAR2 with niacin or BHBA promoted Ca^2+^ mobilization in neutrophils and mononuclear cells. Mononuclear cells treated with BHBA had diminished intracellular Ca^2+^ responses when HCAR2 was knocked down by siRNA silencing, indicating Ca^2+^ mobilization was mediated by HCAR2 signaling. Two candidate antagonists for HCAR2, synthesized from niacin (NA-1 and NA-5), were tested; monocytes and neutrophils pre-treated with NA-1 and NA-5 had reduced Ca^2+^ mobilization after incubation with BHBA. Furthermore, NA-5 but not NA-1 prevented BHBA-associated reductions in cyclic AMP.

**Conclusions:**

We demonstrated that HCAR2 is present on bovine leukocytes and has greater expression later in lactation. We confirmed that BHBA and niacin derived HCAR2 antagonists alter bovine leukocyte activity. Our results demonstrate that both BHBA and niacin affect bovine leukocyte Ca^2+^ mobilization in a HCAR2-dependent manner.

**Supplementary Information:**

The online version contains supplementary material available at 10.1186/s40104-024-00999-5.

## Background

Basal plasma concentrations of BHBA in lactating cows range from 0.3 to 0.7 mmol/L; however, in negative energy balance, liver ketogenesis results in significantly greater plasma BHBA concentrations. Subclinical ketosis is usually defined as a plasma BHBA concentration exceeding 1.2 mmol/L, and concentrations can exceed 3.5 mmol/L in clinical ketosis [1]. The estimated incidence rate of subclinical ketosis in lactating dairy cattle is 15%–18% [2, 3], and the incidence of clinical ketosis is approximately 4% [4].

In lactating dairy cows, the etiology of ketosis is complicated by the fact that it is usually accompanied by depressed feed intake and hepatic steatosis. Also, it is associated with other disorders that are common in early lactation [5]. Cows experiencing ketosis are at greater risk of developing mastitis, metritis, displaced abomasum, and lameness [6]. They are also at greater risk of culling or leaving the herd [1]. In addition to epidemiological links between ketosis and disease, studies have demonstrated that ketones directly impair several immune cell functions such as phagocytosis, oxidative burst, and pathogen killing by neutrophils [7–10] and viability and phagocytic capacity of macrophages [11]. Inducing hyperketonemia in dairy cows seems to promote tolerance during an intramammary mastitis challenge [12]. Additional research indicates that infusing BHBA may reduce somatic cell score (SCS) during an intramammary lipopolysaccharide (LPS) challenge, indicating potential effects of BHBA on cell migration in dairy cattle [13].

The discovery of HCAR2, a G-protein coupled receptor with a high affinity for BHBA, presents a mechanism by which BHBA influences cellular function [14]. Like all G-protein coupled receptors (GPR), HCAR2 (also known as HM74A, GPR109A, and PUMA-G) activates intracellular signaling pathways through adapter proteins [15]. Much of the research on HCAR2 has focused on adipocytes, where its activation decreases cyclic AMP (cAMP) concentrations and subsequently suppresses lipolysis by reducing activity of hormone sensitive lipase [14]. Even though BHBA is considered to be the endogenous ligand for this receptor, it was originally described as a receptor for niacin [16], thus explaining the potent anti-lipolytic effects of niacin.

The role of this receptor in normal immune function is unknown. Kostylina et al. [17] made the critical discovery that niacin promotes apoptosis of neutrophils through HCAR2. Niacin also prevented the recruitment of macrophages stimulated by monocyte chemotactic protein-1 in a HCAR2-dependent manner [18]. Increased neutrophil apoptosis and decreased macrophage migration are consistent with impaired immune function associated with ketosis in dairy cows and observed in disease challenge scenarios [13]. The objective of this study was to clarify whether BHBA mediates bovine leukocyte activity in a HCAR2-dependent manner, determine which immune cells that HCAR2 is expressed on, whether expression of HCAR2 changes with stage of lactation, and whether HCAR2-mediated signaling could be blocked by antagonist compounds derived from niacin.

## Methods

### Flow cytometry

**Fig. 1:**
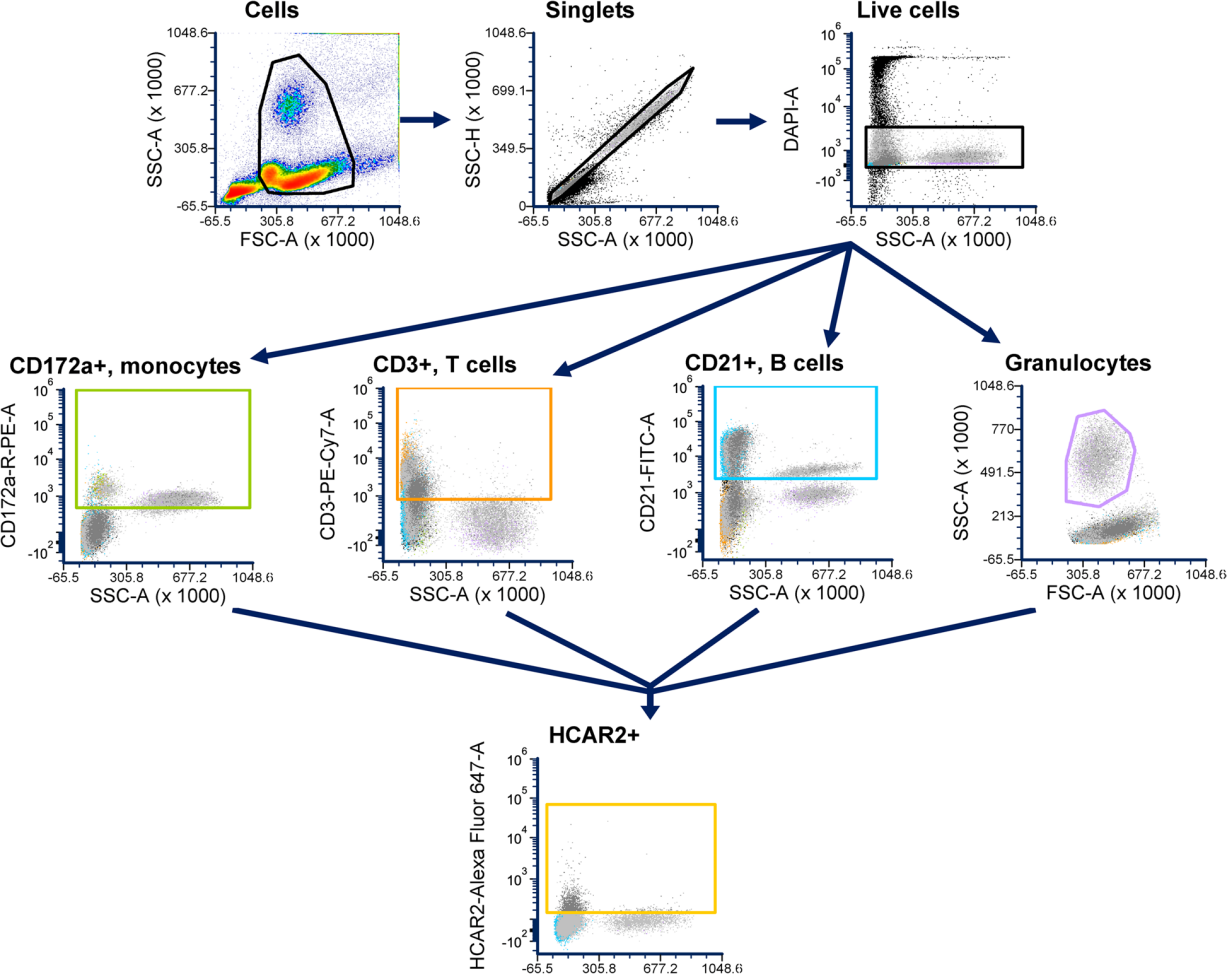
Depiction of gating strategy used for flow cytometric analysis of bovine circulating immune cells. First, debris were gated out, then we captured single cells and stained for live cells. Then, we set gates for each cell population of interest (CD3^+^, CD21^+^, CD172a^+^, and granulocytes) to investigate HCAR2 expression within each population

Blood samples were collected from 6 cows in the first 10 d in milk (DIM; mean ± SD; 4.7 ± 3 DIM) and 6 mid-lactation cows (115–200 DIM; 152 ± 23 DIM) for flow cytometric analysis of circulating immune cells. All cows selected had not experienced clinical disease in the 3 months prior to blood collection. Breifly, blood was collected by coccygeal venipuncture into tubes containing K_2_EDTA and placed immediately on ice for transport back to the lab. Samples were centrifuged for 15 min at 1,500× *g* at 4 °C to remove the plasma. Then, 5 mL of cold Ca^2+^Mg^2+^-free DPBS were added, the contents were mixed, and centrifuged for 10 min at 1,500× *g* at 4 °C. The supernatant was removed and 8 mL of ACK lysis buffer were added; the sample was inverted twice and then the sample was incubated at 4 °C in the dark for 8 min to lyse red blood cells. Next, the sample was centrifuged for 10 min at 400× *g* at 4 °C. The supernatant was removed and 8 mL of cold Ca^2+^Mg^2+^-free DPBS was added and the cell pellet was resuspended and centrifuged for 5 min at 400× *g* at 4 °C. The supernatant was then discarded. This washing step was repeated once more. After final wash, the cell pellet was resuspended in 1 mL Ca^2+^Mg^2+^-free DPBS. Cell suspensions were aliquoted (100 µL/well) into a 96-well plate along with 100 µL of Ca^2+^Mg^2+^-free DPBS. Cells were washed and then incubated with 50 µL of FBS for 10 min in the dark. Then, cells were stained with 50 µL of antibody mastermix cocktail which is described in Supplemental Table 1. Antibodies used in this panel were CD172a (HR-BOV2049, WSU Antibody Center, Pullman, WA, USA) conjugated to PE/R-PE (abcam102918, Cambridge, UK); CD3 (Bu-BOV2009, WSU Antibody Center) conjugated to PE-Cy7 (ab102903, Cambridge, UK); CD21 with FITC fluorophore (MCA1424F, Bio-Rad); and HCAR2 with AlexaFluor 647 (51-4779-42, ThermoFisher Scientific). Cells were washed twice, resuspended in FACS buffer, and stained with 4´,6-diamidino-2-phenylindole, dilactate (DAPI; D3571, ThermoFisher Scientific) immediately prior to cytometric analysis using an Attune CytPix benchtop brightfield-imaging capable analyzer (ThermoFisher Scientific). The general gating scheme can be found in Fig. 1. All gates were set conservatively using fluorescence-minus-one controls.

### Leukocyte isolation, HCAR2 functionality, and HCAR2 silencing

We isolated peripheral blood mononuclear cells (PBMC) and polymorphonuclear leukocytes (PMN) from lactating dairy cattle. Heparinized blood (180 mL) was collected from 6 healthy Holstein cows in mid-lactation [19] and diluted with Ca^2+^, Mg^2+^ free PBS at a 1:1 ratio. The PBMC were separated from whole blood by centrifugation using Ficoll-Paque PLUS (GE Healthcare, Little Chalfont, UK) at 800× *g* for 30 min at 4–8 °C. After centrifugation, serum was removed and PBMC were collected and washed 3 times with Hanks balanced salt solution (Sigma-Aldrich Chemical Co., St. Louis, MO, USA). The pellet containing the erythrocytes and PMN was treated twice with hypotonic phosphate-buffered deionized water and restored with hypertonic phosphate-buffered 2.7% NaCl to lyse the erythrocytes. The PMN were pelleted by centrifugation at 400× *g* for 5 min. For both cell types, viability was determined by Trypan Blue exclusion. Cells were resuspended in RPMI 1640 medium containing 10% FBS and 1% Pen/Strep at 1.5 × 10^6^ cells/mL and were plated in 96-well black flat-bottom plates.

To assess whether the HCAR2 expressed by leukocytes was functional, we treated primary bovine neutrophils and mononuclear cells with niacin, a potent agonist for this receptor, and with BHBA, the endogenous ligand. We used a niacin concentration (10 µmol/L) reported to induce calcium mobilization in human neutrophils [17] and a dose of BHBA (1 mmol/L) that is slightly above its reported EC_50_ [15] but well within the physiological range in dairy cattle. Calcium mobilization assays were conducted using the Fluo-4 Direct Calcium assay kit (Life Technologies). Isolated PBMC and PMN were plated in 96-well black flat-bottom plates and treated with BHBA and niacin.

Cyclic AMP was measured using a chemiluminescent immunoassay kit according to manufacturer instructions (cat# 4412183; Applied Biosystems). Forskolin (J63292.MA, ThermoFisher Scientific), which activates adenyl cyclase and consequently increases cAMP concentrations [20], was used as a positive control.

To determine if HCAR2 facilitates the Ca^2+^ response to BHBA, siRNA targeting bovine HCAR2 (target sequence 5´-AACAAGATCTCCAATCGGACA-3´; custom siRNA from ThermoFisher Scientific) and control siRNA Silencer™ (Negative Control No. 2 siRNA AM4613 from ThermoFisher Scientific) were designed. Although PMN are of great interest, PBMC have greater cell viability in culture and are therefore more amenable to RNA silencing techniques. The PBMC were isolated as described above, resuspended in RPMI 1640 medium containing 10% FBS, 1% Pen/Strep (1.5 × 10^6^ cells/mL), plated in 12- or 96-well plates, and incubated at 37 °C in 5% CO_2_. After 2 h, cells were transfected with 10 nmol/L siRNA (control and HCAR2) in serum-free medium with siPORT NeoFX Transfection Agent (AM4511, ThermoFisher Scientific) and incubated for 24 h. Cells were then treated with 1 mmol/L BHBA and Ca^2+^ mobilization was assessed, or cells were harvested to determine knockdown efficiency. HCAR2 knockdown was confirmed by Western blot.

### HCAR2 protein expression

Isolated cell protein was recovered after lysis of cells with RIPA lysis buffer (ThermoFisher Scientific, Carlsbad, CA, USA) with protease inhibitor added (Protease inhibitor cocktail I; Calbiochem, Gibbstown, NJ, USA). The homogenate was centrifuged at 15,000× *g* for 10 min at 4 °C, and total protein concentration of the supernatant was measured (BCA Protein Assay kit, ThermoFisher Scientific). Forty micrograms of total protein were separated by SDS-PAGE on a 4%–12% Tris–HCl gel and dry-transferred onto nitrocellulose membranes (iBlot; Invitrogen Corp.). Membranes were blocked in Tris buffer (pH 7.4) with 5% dry milk powder for 1 h at room temperature, then incubated with a rabbit anti-HCAR2 IgG (PA5-90579 diluted 1:1,000; Invitrogen, ThermoFisher Scientific) for 1 h at room temperature. After washing, membranes were incubated for 1 h at room temperature with a secondary goat anti-rabbit IgG (7074S; Cell Signaling Technology, Beverly, MA, USA) diluted 10,000-fold in Tris buffer (pH 7.4). Immunodetection was performed by chemiluminescence (West-Dura; ThermoFisher Scientific), and band images were visualized using a photo documentation system (ChemiDoc-It Imaging System; Bio-Rad, Hercules, CA, USA).

### Fragment-based drug discovery/molecular docking

**Fig. 2:**
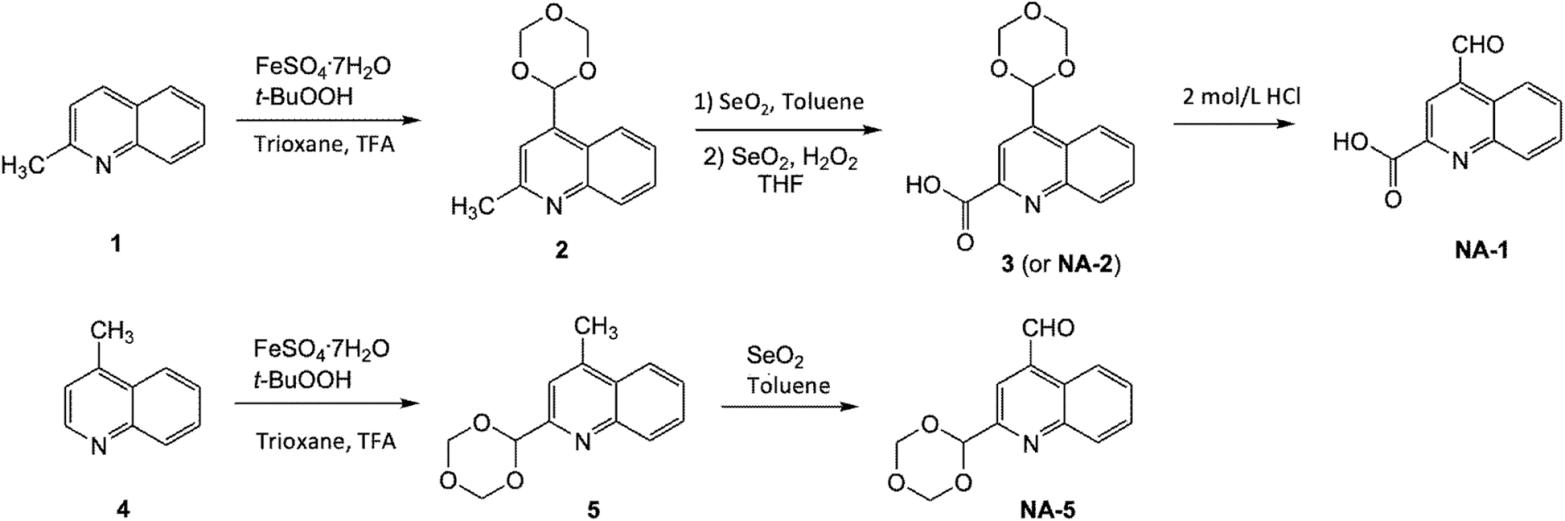
Synthesis of NA-1 and NA-5

To search for HCAR2 antagonists, we conducted computational docking experiments utilizing a fragment-based drug discovery approach [21]. We generated a library of compounds focusing on substituted quinoline molecules (code name NA). This class of molecules possesses the pyridine-like nucleus and oxygen moiety of the active agonist, niacin. Representative molecules such as NA-1 and NA-5 were synthesized from 2-methylquinoline (**1**) and 4-methylquinoline (**4**), respectively, as depicted in Fig. 2. In brief, trioxanylation of **1** with 4 mol% of ferric sulfate heptahydrate, *t*-butyl hydroperoxide, trioxane, trifluoroacetic acid (TFA) in acetonitrile gave 4-trioxanyl **2** [22, 23]. Benzylic oxidation of **2** with selenium dioxide in toluene followed by oxidation of the resulting carboxaldehyde with selenium dioxide and hydrogen peroxide gave compound **3** or NA-2. Hydrolysis of the trioxanyl moiety with hydrochloric acid afforded NA-1. Similarly, trioxanylation of **4** followed by benzylic oxidation furnished NA-5. More thorough descriptions can be found in Additional file 1. NA-1 and NA-5 were then tested for the ability to inhibit the activation of HCAR2 by BHBA using the calcium mobilization assay, as previously described.

### Cell viability assay

Isolated PMN were treated with BHBA (1 mmol/L), NA-1 (100 µmol/L), NA-5 (100 µmol/L), BHBA + NA-1, or BHBA + NA-5, then incubated for 4 h at 37 °C in 5% CO_2_. Afterwards, alamarBlue reagent (ThermoFisher Scientific) was added to wells and further incubated for 4 h at 37 °C in 5% CO_2_. Cell viability was assessed by measuring absorbance at 570 nm using a plate reader (Synergy HTX; BioTek Instruments Inc., Winooski, VT, USA) and Gen5 software (BioTek Instruments Inc.).

### Statistical analysis

Data were analyzed with ANOVA using R (version 4.03). Analysis of flow cytometry data included the fixed effect of stage of lactation (early vs. late). The Ca^2+^ mobilization model included the effects of cell type, treatment, and their interaction. Log_10_-transformed cAMP data were analyzed using a mixed model approach which included the random effect of cell culture plate in addition to the fixed effect of treatment. Pairwise differences were adjusted using Tukey or Sidak adjustments. Significance was declared at *P* ≤ 0.05 and trends were declared at 0.05 ≤ *P* ≤ 0.10. Normality of residuals were evaluated visually and using Shapiro-Wilks test. Observations with Studentized residuals > 3 or < –3 were considered outliers and removed; observations were also removed if Cooks *D* was > 0.5.

## Results

**Fig. 3:**
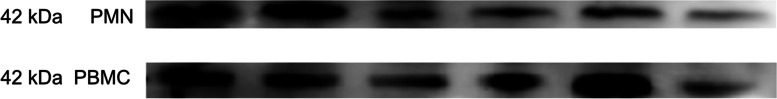
HCAR2 protein expression on bovine polymorphonuclear cells (PMN) and peripheral blood mononuclear cells (PBMC) from 6 healthy mid-lactation Holstein cows

A robust expression of HCAR2 was found at the protein level in PBMC and PMN (Fig. 3). Also, HCAR2-expressing cells tended to be greater in mid-lactation compared to early lactation (*P* = 0.09; Table 1) with a greater proportion of B-cells expressing HCAR2 in mid-lactation (*P* < 0.01). T-cells also tended to have a greater proportion of cells expressing HCAR2 in mid-lactation (*P* = 0.09).

**Table 1:** Expression of HCAR2 on circulating bovine immune cell populations in early and mid-lactation dairy cows (*n* = 6 per stage of lactation)

Item^1^	Early lactation^2^	Mid-lactation	SEM	*P*-value
HCAR2^+^, % of live cells	1.71	3.53	0.849	0.09
Monocytes, % of live cells	30.03	32.82	6.715	0.75
HCAR2^+^, % of Monocytes	1.90	1.83	0.859	0.95
T-cells, % of live cells	24.26	28.97	4.005	0.34
HCAR2^+^, % of T-cells	3.24	5.37	1.030	0.09
B-cells, % of live cells	37.88	38.04	5.255	0.98
HCAR2^+^, % of B-cells	2.12	5.44	0.811	< 0.01
Granulocytes, % of live cells	26.60	28.51	6.883	0.84
HCAR2^+^, % of granulocytes	1.00	1.52	0.468	0.33

^1^Monocytes = CD172a^+^, B-cells = CD21^+^, T-cells = CD3^+^, Granulocytes separated by side scatter. Cell populations do not add to 100% due to some B-cells being counted as granulocytes; this may be related to bovine leukemia virus present in this population of cows

^2^Early lactation cows were less than 10 d in milk and mid-lactation cows were between 115 and 200 d in milk. No cows had adverse health events in the 90 d prior to sampling

**Fig. 4:**
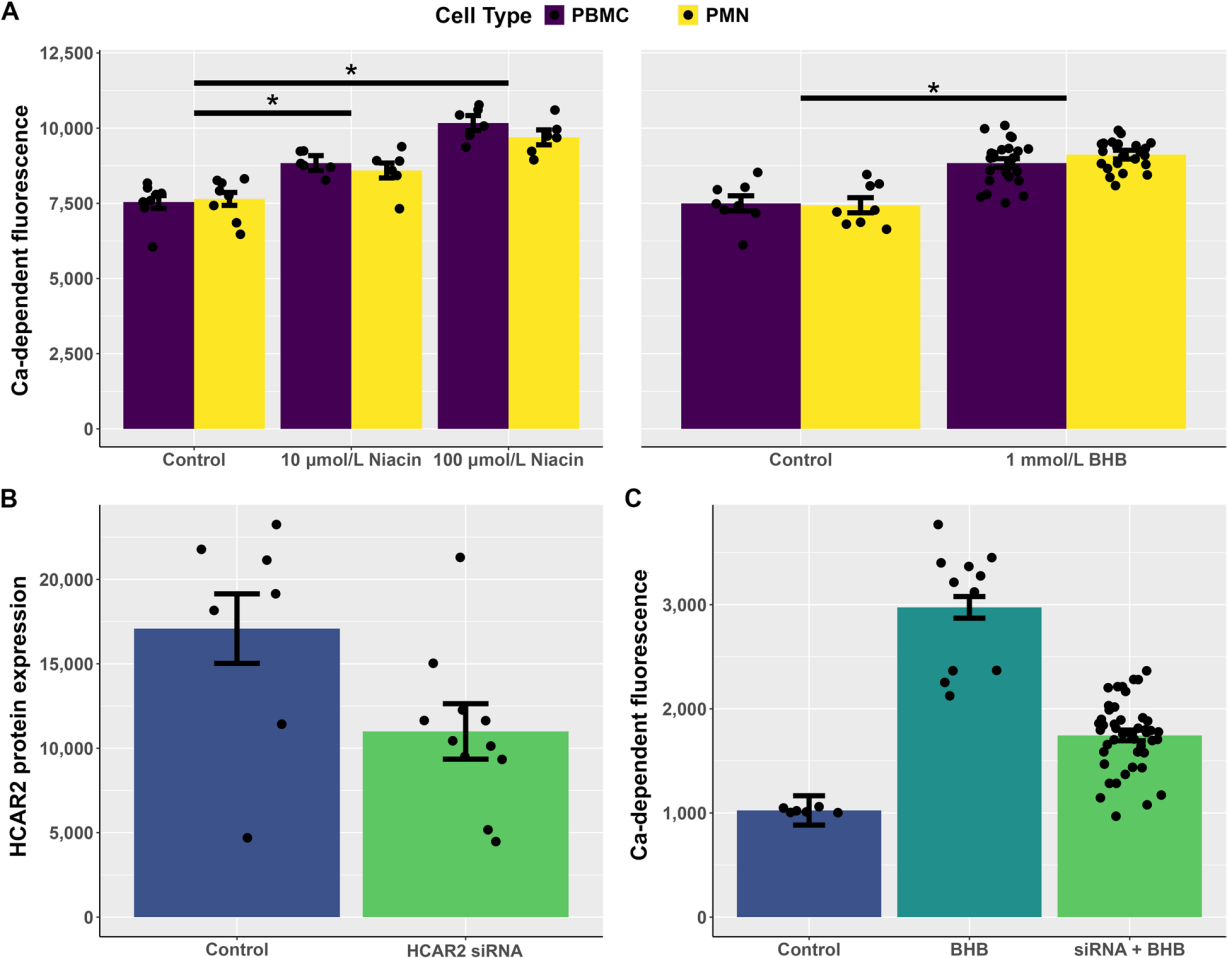
**A** Calcium flux of neutrophils and monocytes stimulated with 10 and 100 μmol/L of niacin or 1 mmol/L of BHBA. Both 10 and 100 μmol/L of niacin increased calcium flux relative to the control (*P* < 0.01). BHBA also increased calcium flux relative to the control (*P* < 0.01). **B** HCAR2 protein expression on bovine monocytes with and without siRNA knockdown. siRNA reduced HCAR2 protein expression by 36% (*P* = 0.03). **C** Calcium flux of monocytes with or without 1 mmol/L of BHBA and in the present of siRNA. BHBA increased calcium flux (*P* < 0.01) whereas siRNA in the presence of BHBA reduce calcium flux by 41% (*P* < 0.01)

Incubating either PMN or PBMC with niacin or BHBA increased (*P* < 0.01) cytosolic Ca^2+^ (Fig. 4A). Niacin was a more potent ligand, as 10 µmol/L and 100 µmol/L niacin increased Ca^2+^ by 12% and 27%, respectively, whereas 1 mmol/L BHBA increased Ca^2+^ by 23%. Using siRNA, we knocked down HCAR2 in PBMC and achieved 36% reduction in HCAR2 protein after 24 h (Fig. 4B, Additional file 1: Supplemental Fig. 1). We observed that siRNA knockdown of HCAR2 reduced Ca^2+^ concentrations 42% when PBMC were incubated with BHBA (Fig. 4C).

**Fig. 5:**
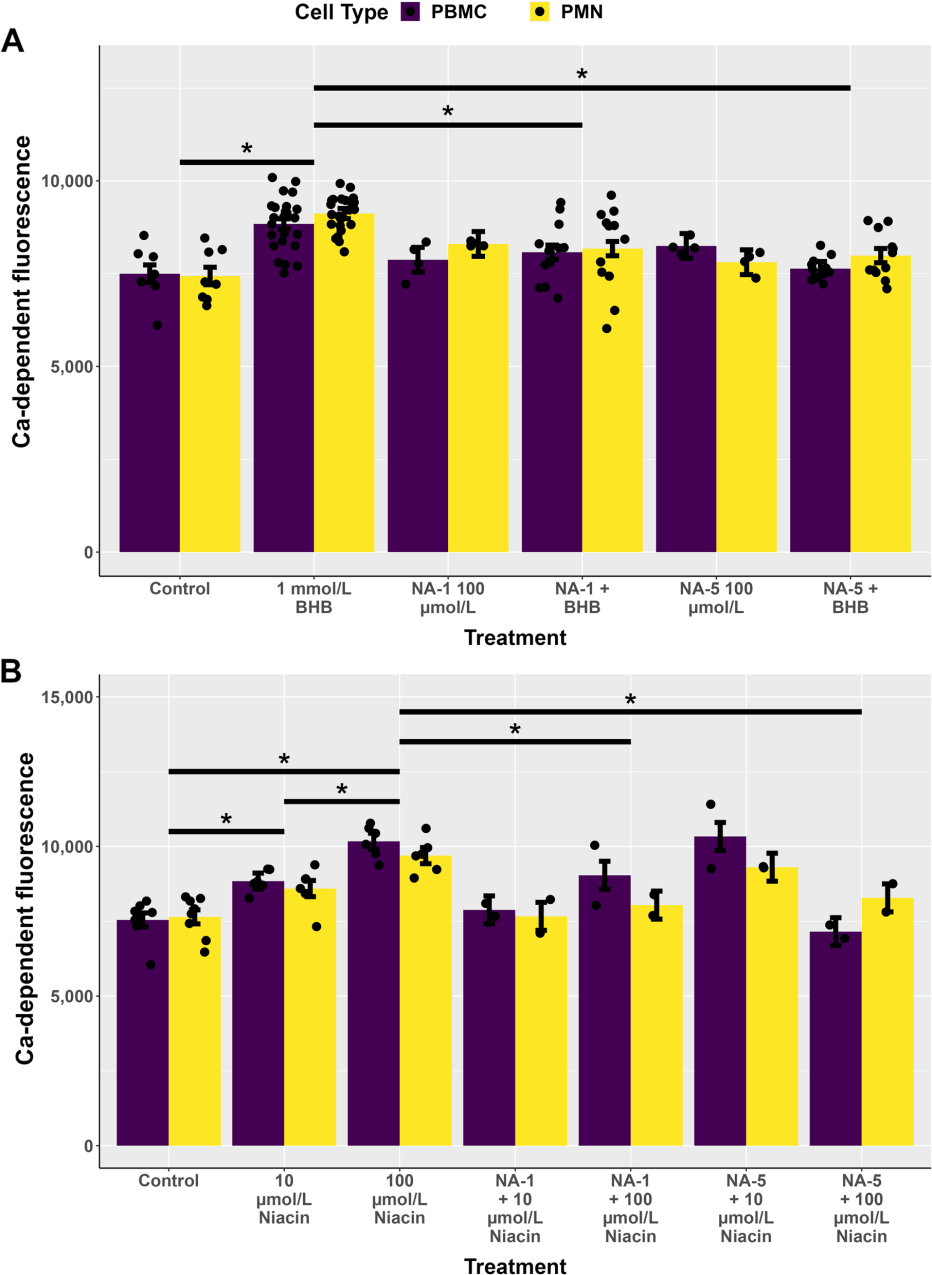
**A** Beta-hydroxybutyric acid (BHBA) increased Ca-dependent fluorescence (*P* < 0.01) whereas NA-1 and NA-5 reduced Ca-dependent fluorescence in the presence of BHBA (*P* < 0.01). **B** 10 and 100 μmol/L niacin increased Ca-dependent fluorescence (*P* < 0.01). NA-1 and NA-5 reduced calcium fluorescence in the presence of 100 μmol/L niacin (*P* ≤ 0.01)

In an attempt to mitigate the activation of HCAR2 by either niacin or BHBA, we generated 10 niacin-derived antagonists (NA-1 to NA-10); NA-1 and NA-5 were selected as suitable candidates (Fig. 5A) for further evaluation. The addition of NA-1 or NA-5 did not increase Ca^2+^ concentrations in PBMC or PMN (Fig. 5B). When cultured in the presence of 1 mmol/L BHBA, both NA-1 and NA-5 reduced Ca^2+^ concentrations (Fig. 5B). NA-1 and NA-5 also reduced Ca^2+^ concentrations when incubated with 100 µmol/L niacin (Fig. 5C).

**Fig. 6:**
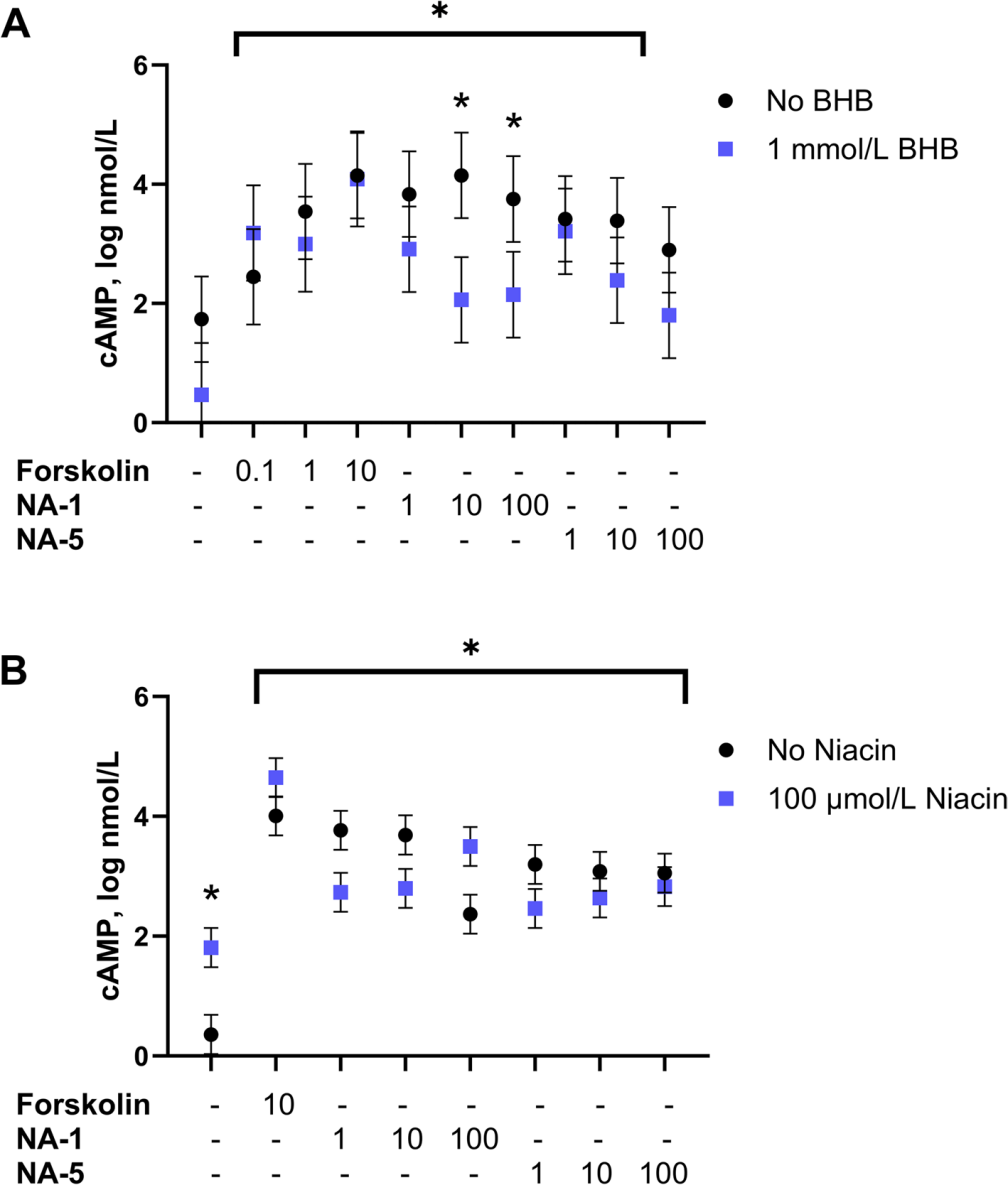
Log_10_-transformed cAMP concentrations of PMN cells. Forskolin (FSK; 0.1, 1, or 10 μmol/L) served as the positive control to increase cAMP. No FSK, NA-1, or NA-5 was the negative control (far left column). Treatments under the horizontal line were different from negative control (*P* ≤ 0.05). Asterisks within a column indicate effects of niacin or BHBA on cAMP (*P* ≤ 0.05). **A** BHB reduced cAMP when incubated with 10 or 100 μmol/L of NA-1. **B** Niacin increased cAMP but did not affect cAMP concentrations when incubated with NA-1 or NA-5

Cyclic AMP concentrations were increased when PMN were incubated with forskolin (*P* < 0.01) which served as the positive control (Fig. 6A, B). Addition of NA-1 and NA-5 in the absence of BHBA or niacin increased cAMP concentrations compared to the control. BHBA reduced cAMP when incubated with 10 and 100 µmol/L NA-1 but not when incubated with NA-5 (Fig. 6A). Niacin alone (100 µmol/L) increased cAMP but there was no difference in response when incubated with NA-1 or NA-5 (Fig. 6B).

## Discussion

HCAR2 has been characterized as a metabolic sensor, modulating cell signaling associated with energy metabolism and inflammatory signaling [24]. Its presence in bovine tissues [25] has increased interest in HCAR2’s role in immunosuppression observed during ketosis. In agreement with the current study, the presence of HCAR2 in bovine leukocytes has been reported previously [26, 27]. To our knowledge, this is the first investigation of HCAR2 expression in monocytes, lymphocytes, and granulocytes across different stages of lactation. We observed that the proportion of cells expressing HCAR2 tends to be greater later in lactation, with expression in lymphocytes driving that difference. Since HCAR2 has anti-inflammatory effects, its reduced expression earlier in lactation may contribute to inflammation observed in dairy cattle after calving [28]. Interestingly, the very low expression on granulocytes would suggest that ketosis would have smaller effects on the innate immune response and larger effects on the adaptive response. Stimulation by LPS, TNF-α or IL-1β increased the expression of HCAR2 in mice in both adipocytes and macrophages [29], and the healthy status of the cows sampled in this study may have contributed to the relatively low proportions of HCAR2^+^ cells we observed here. Understanding how cell activation regulates HCAR2 expression in bovine leukocytes, especially during the postpartum period, may be an intriguing area of future investigation and help explain the connection between ketosis, inflammation, and disease.

Both niacin and BHBA activate HCAR2 in human and mouse immune cells [30, 31]. HCAR2 ligands, with particular emphasis on BHBA, affected bovine neutrophils harvested from milk and blood [9–11, 32]. In cells of the immune system, Gi-activation via Gβγ-subunits leads to an activation of β-isoforms of phospholipase C [33]. The activation of the pathway promotes an increase in intracellular Ca^2+^ concentration [34, 35] which aligns with our results, as both niacin and BHBA increased intracellular Ca^2+^.

Niacin administrated increases intracellular Ca^2+^ in mouse macrophages in vivo [36] and human neutrophils in vitro [17] in an HCAR2-dependent manner, which agrees with our observations and others in bovine cells and murine RAW264.7 cells [32, 37]. Similar findings were observed in human carcinoma cells, where HCAR2 activation by niacin induced intracellular Ca^2+^ release; this effect was abolished by a pretreatment with a G-protein coupled receptor inhibitor—indicating a Gi-dependent mechanism [38]. Furthermore, our results indicated that intracellular Ca^2+^ flux was dependent on HCAR2, as siRNA knockdown attenuated the Ca^2+^ response observed when cells were incubated with BHBA or niacin.

HCAR2 agonists have exhibited immune cell modulating effects such as decreased leukocyte migration [39]. This is also supported in bovines as ketones have reduced the oxidative burst, phagocytosis, and migratory capacity of leukocytes [9–11]. Ketones and niacin also affect the mitogenic capacity of lymphocytes [40]. However, Carretta et al. [32] observed that both BHBA and niacin supplementation in vitro increased chemoattractant function of neutrophils harvested from heifers. The effect of HCAR2 agonists on immune cell migration is also inconsistent in vivo as BHBA infusions have reduced or not affected SCS in mastitis challenge models [12, 13]. Possibly, the physiological status (lactating vs. non-lactating; stage of lactation) or the disease challenge model (LPS vs. *S. uberis*) may affect the response of leukocytes to BHBA. Regulatory mechanisms in the animal likely aim for a balanced response; reducing immune cell functions such as migration and oxidative burst may reduce excess inflammation and associated pathology, but as observed in ketotic cows, it may render them more susceptible to infectious disease. Additionally, further investigation of HCAR2’s effects on cytokine production in bovines is warranted. Niacin reduced production of TNF-α, IL-1β, and IL-6 in RAW264.7 murine macrophages [31] but that has not been observed in bovine cells. Our observation of increased Ca^2+^ would suggest that similar results may be observed for bovines.

Dampened immune cell response has been associated with several health disorders in dairy cows. For instance, reduced neutrophil function is associated with greater risk of retained placenta and metritis [41, 42] and depressed macrophage function may increase the risk of clinical mastitis and interdigital dermatitis [43]. Therefore, inhibiting BHBA activation of HCAR2 could improve leukocyte function and their capacity to respond to tissue injury or pathogens.

The fragment-based approach to drug discovery has been established as an efficient tool in the search for new drugs, involving the construction of potent small-molecule ligands from low-molecular mass fragment molecules [21]. In the current study, two compounds (NA-1 and NA-5) from the initial library appeared to be effective in inhibiting the activation of HCAR2 by BHBA and niacin as demonstrated by the Ca^2+^ mobilization assay. In contrast, both NA-1 and NA-5 increased cAMP above control, and in the presence of BHBA, NA-1 reduced cAMP production. Interestingly, HCAR2 activation in immune cells may augment cAMP production, which contrasts reductions in cAMP observed in adipocytes incubated with HCAR2 agonists [44]. Our data offers some insight into this differential cAMP response for bovine cells; when niacin was incubated with PMN, it increased cAMP production, whereas BHBA did not change cAMP. Furthermore, it may be that NA-1 and NA-5 were partial or competitive agonists for HCAR2, as they increased cAMP production compared to control. Further investigation of cell-specific responses to HCAR2 agonists in bovine cells would be fruitful.

Systemically blocking HCAR2 in transition cows could enhance the innate immune response, but it could also promote uncontrolled lipolysis and consequently increase circulating free fatty acid and ketone body concentrations. The activation of HCAR2 in adipose tissue exerts an anti-lipolytic effect [29] and blocking it may promote unregulated lipolysis. Studies have suggested that free fatty acid concentrations in plasma are negatively associated with immune function [45, 46] but no effects of increasing concentrations of free fatty acids were observed on phagocytosis capacity and apoptosis in vitro. Free fatty acids even increased oxidative burst in bovine PMN [47]. On the other hand, BHBA exposure (at concentrations like those experienced during ketosis) has consistently impaired phagocytosis, microbial killing, and various antimicrobial mechanisms of neutrophils from bovine milk and blood [9–11, 48]. Reduced migration of leukocytes due to elevated BHBA has also been demonstrated in vivo [13] during an LPS mastitis challenge. Furthermore, neutrophil infiltration was reduced in an LPS induced mastitis model in which mice were supplemented with niacin [49]. Reduced migration and inflammation by activating HCAR2 may be positive as it may reduce excess inflammation and pathology associated with diseases like mastitis.

## Conclusions

This study revealed that bovine leukocytes express HCAR2 and that the proportion of HCAR2 cells was greater later in lactation. Also, Ca^2+^ mobilization is increased with BHBA or niacin activation of HCAR2. In addition, we screened niacin-derived antagonists and found that NA-1 and NA-5 inhibited Ca^2+^ mobilization. BHBA reduced cAMP concentrations when incubated with NA-1 but not NA-5. Further research should investigate whether NA-1 or NA-5 reduce effects associated with HCAR2 ligands on immune cell functions such as phagocytosis, chemotaxis, and oxidative burst.

### Supplementary Information

**Additional file 1: Supplemental Methods. **Detailed Synthesis of NA-1 and NA-5. **Supplemental Table 1.** Antibody cocktail used to measure the expression of HCAR2 in circulating bovine immune cells. **Supplemental Fig. 1.** HCAR2 protein expression on bovine peripheral mononuclear cells (PBMC) treated with control siRNA or HCAR2 siRNA for 24 h. Example lanes shown are representative of 7 control and 11 HCAR2 siRNA replicates.

## Data Availability

The datasets used and/or analyzed during the current study are available from the corresponding author upon reasonable request. Procedures for the syntheses of NA-1 and NA-5 are described in Additional file 1.

## Abbreviations

BHBA: Beta-hydroxybutyric acid
cAMP: Cyclic AMP
FBS: Fetal bovine serum
GPR: G-protein coupled receptor
NA: Synthesized HCAR2 antagonists derived from nicotinic acid
PBMC: Peripheral blood mononuclear cells
PBS: Phosphate buffer saline
PMN: Polymorphonuclear leukocytes
