# Supplementary material for: Investigation of HCAR2 antagonists as a potential strategy to modulate bovine leukocytes
Source: J Anim Sci Biotechnol. 2024 Mar 6;15:38. doi: 10.1186/s40104-024-00999-5 (PMC10916251; doi:10.1186/s40104-024-00999-5)
Supplement: Supplementary file 1 — Additional file 1: Supplemental Methods. Detailed Synthesis of NA-1 and NA-5. Supplemental Table 1. Antibody cocktail used to measure the expression of HCAR2 in circulating bovine immune cells. Supplemental Fig. 1. HCAR2 protein expression on bovine peripheral mononuclear cells (PBMC) treated with control siRNA or HCAR2 siRNA for 24 h. Example lanes shown are representative of 7 control and 11 HCAR2 siRNA replicates. [file 40104_2024_999_MOESM1_ESM.pdf]

***Short Report:* Investigation of HCAR2 antagonists as a potential strategy to prevent hyperketonemia associated dysfunction in bovine leukocytes.**

L. K. Mamedova, K.C. Krogstad, Laxman Pokhrel, Duy H. Hua, E. C. Titgemeyer, and B. J. Bradford

***Supplemental Methods: Detailed Synthesis of NA-1 and NA-5*.**

2-Methyl-4-(1,3,5-trioxan-2-yl)quinoline (**2**). To a mixture of 10.0 g (70 mmol) of 2-methylquinoline (**1**) and 9.5 g (84 mmol) of trifluoroacetic acid (TFA) were added 20 mL (0.28 mol) of 70% *t*-butyl hydroperoxide, 0.78 g (2.8 mmol) of $FeSO_4 \cdot 7H_2O$, 160 g of trioxane, and 160 mL of acetonitrile. The reaction solution was heated to reflux for 12 h, cooled to 25ºC, basified with 5% aqueous NaOH, and extracted three times with diethyl ether. The combined organic layer was washed with water and brine, dried (anhydrous Na_2SO_4), concentrated and column chromatographed on silica gel using a gradient mixture of hexane and ethyl acetate as eluent to give 5.3 g (51% yield based on recovered **1**) of compound **2** along with 3.5 g of recovered **1**. Compound **2**: ^{1}H NMR (400 MHz in $CDCl_3$) δ 8.05 (d, J = 8.6 Hz, 2H), 7.66 (t, J = 7.8 Hz, 1H), 7.55 (s, 1H), 7.49 (t, J = 7.8 Hz, 1H), 6.33 (s, 1H), 5.44 – 5.36 (m, 4H), 2.73 (s, 3H); ^{13}C NMR (100 MHz in $CDCl_3$) δ 159.0, 148.4, 140.4, 129.4, 129.39, 126.1, 123.6, 123.3, 119.5, 98.8, 93.8, 25.5; MS (electrospray ionization) m/z 232.2 ($M+H^+$), 172.0, 144.1.

4-Formylquinoline-2-carboxylic acid (NA-1). A solution of 0.70 g (3.0 mmol) of **2** and 0.34 g (3.0 mmol) of SeO_2 in 30 mL of toluene, under argon, was stirred at 80ºC for 15 h. The reaction mixture was diluted with dichloromethane, washed with brine, dried ($MgSO_4$), concentrated, and column chromatographed on silica gel using a gradient mixture of hexanes and ethyl acetate as eluent to give 0.60 g (81% yield) of 4-(1,3,5-trioxan-2-yl)quinoline-2-

carboxaldehyde: ^{1}H NMR (400 MHz in $CDCl_3$) δ 10.24 (s, 1H, CHO), 8.32 (d, *J* = 9.0 Hz, 2H), 8.31 (s, 1H), 7.85 (ddd, *J* = 8.5, 7.1, 1.2 Hz, 1H), 7.75 (ddd, *J* = 8.4, 6.6, 1.2 Hz, 1H), 6.47 (s, 1H), 5.51 – 5.46 (m, 4H); ^{13}C NMR (100 MHz in $CDCl_3$) δ 193.5 (CHO), 152.5, 148.6, 142.1, 131.3, 130.6, 129.8, 127.0, 124.6, 115.5, 99.1, 93.9; MS (electrospray ionization) m/z 267.9 (100%) ($M+Na^+$), 245.9 ($M+H^+$). To a solution of 0.60 g (2.4 mmol) the above aldehyde in 7 mL of THF were added 27 mg (0.24 mmol) of SeO_2 and 3.5 mL of 30% H_2O_2. The solution was stirred at 40ºC for 3 h, cooled to 25ºC, diluted with 20 mL of water, acidified with 2N HCl to pH ~1, and extracted with ethyl acetate three time. The combined organic layer was washed with brine, dried ($MgSO_4$), and concentrated to give 0.47 g (75% yield) of carboxylic acid **3**. A solution of 0.47 g of **3** in 150 mL of 2N HCl was heated to reflux for 3 h, cooled to 25ºC, neutralized with aqueous K_2CO_3 to pH ~5, and extracted with dichloromethane three times. The combined organic layer was washed with water and brine, dried (anhydrous Na_2SO_4), concentrated and column chromatographed on silica gel using a gradient mixture of hexane, dichloromethane and methanol as eluents to give 0.29 g (80% yield) of NA-1: ^{1}H NMR (400 MHz in $CDCl_3$) δ 10.6 (s, 1H, CHO), 9.17 (d, *J* = 9.0 Hz, 1H), 8.67 (s, 1 H), 8.30 (d, *J* = 9 Hz, 1H), 7.90 (m, 2H); ^{13}C NMR (100 MHz in $CDCl_3$) δ 192.1 (CHO), 163.8 (CO_2H), 147.7, 146.3, 139.4, 132.1, 131.8, 130.1, 125.8, 125.5, 124.6; HRMS calcd for $C_{11}H_7NO_3^+$ (M^+) 201.0426, found 201.0437.

4-Methyl-2-(1,3,5-trioxan-2-yl)quinoline (**5**). To a mixture of 5.0 g (35 mmol) of 4-methylquinoline (**4**) and 4.75 g (42 mmol) of TFA were added 10 mL (140 mmol) of 70% *t*-butyl hydroperoxide, 0.39 g (1.4 mmol) of $FeSO_4•7H_2O$, 80 g of trioxane and 80 mL of acetonitrile. The reaction solution was heated to reflux for 12 h, cooled to 25ºC, basified with 5% aqueous NaOH, and extracted three times with diethyl ether. The combined organic layer was washed with water and brine, dried (anhydrous Na_2SO_4), concentrated and column chromatographed on silica gel

using a gradient mixture of hexane and ethyl acetate as eluent to give 3.0 g (43% yield) of compound **5**: ^{1}H NMR (400 MHz in $CDCl_3$) δ 8.11 (d, *J* = 8.2 Hz, 1H), 7.92 (d, *J* = 8.5 Hz, 1H), 7.67 (t, *J* = 7.6 Hz, 1H), 7.61 (s, 1H), 7.52 (t, *J* = 7.6 Hz, 1H), 6.04 (s, 1H), 5.41 – 5.33 (m, 4H), 2.66 (s, 3H); ^{13}C NMR (100 MHz in $CDCl_3$) δ 154.9, 147.0, 145.9, 130.1, 129.5, 128.4, 127.0, 123.7, 118.9, 102.2, 93.7, 18.9; MS (electrospray ionization) m/z 270.2 ($M+K^+$), 254.1 ($M+Na^+$), 232.2 ($M+H^+$).

2-(1,3,5-Trioxan-2-yl)quinoline-4-carboxaldehyde (NA-5). A solution of 0.30 g (1.3 mmol) of **5** and 0.19 g (1.7 mmol) of SeO_2 in 5 mL of toluene, under argon, was heated to reflux for 24 h, and cooled to 25ºC. The reaction mixture was diluted with dichloromethane, washed with brine, dried ($MgSO_4$), concentrated, and column chromatographed on silica gel using a gradient mixture of hexanes and ethyl acetate as eluent to give 0.13 g (50% yield based on recovered **5**) of NA-5, as a solid, along with 40 mg of recovery of **5**. NA-5: ^{1}H NMR (400 MHz in $CDCl_3$) δ 10.53 (s, 1H, CHO), 9.08 (dd, *J* = 8.0, 1.2 Hz, 1H), 8.26 (dd, *J* = 7.8, 0.8 Hz, 1H), 8.25 (s, 1H), 7.86 (ddd, *J* = 7.8, 7.0, 1.6 Hz, 1H), 7.78 (ddd, *J* = 7.8, 7.0, 1.6 Hz, 1H), 6.21 (s, 1H), 5.49 – 5.45 (m, 4H); ^{13}C NMR (100 MHz in $CDCl_3$) δ 193.0 (CHO), 155.8, 148.3, 138.0, 130.7, 130.2, 130.1, 124.7, 124.3, 124.0, 101.3, 93.8; MS (electrospray ionization) m/z 268.3 (100%) ($M+Na^+$).

Supplemental Table 1. Antibody cocktail used to measure the expression of HCAR2 in circulating bovine immune cells.

Marker	Source[1]	Host	Isotype	Clone	Fluor	Final well antibody concentration, mg/mL
DAPI	ThermoFisher					5.000
CD172a	WSU		IgG1	HR-DH59B	(R)PE	0.188
CD3	WSU	Mouse	IgG1	MM1A	PE-Cy7	0.188
CD21	BioRad	Mouse	IgG1	CC21	FITC	0.500
HCAR2	ThermoFisher	Mouse	IgG1	4NZBRGO	AF 647	0.125

[1]WSU = Washington State Monoclonal Antibody Center, Pullman, WA.

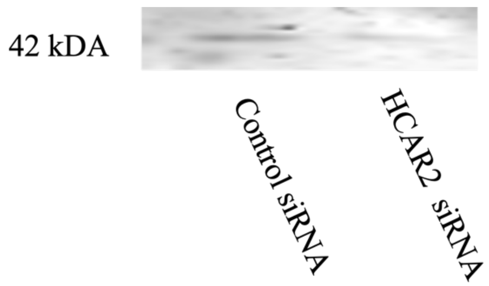

Supplemental Figure 1. HCAR2 protein expression on bovine peripheral mononuclear cells (PBMC) treated with control siRNA or HCAR2 siRNA for 24 h. Example lanes shown are representative of 7 control and 11 HCAR2 siRNA replicates.
